# Affinity Maturation and Characterization of the Novel Monoclonal Antibody (mAb) PB-223 Targeting Cancer-Specific O-Glycans Terminating with α(2,6) Sialic Acids

**DOI:** 10.3390/cancers18142336

**Published:** 2026-07-20

**Authors:** Kwong Y. Tsang, Anjum Zaki, Sharon A. Mavroukakis, Philip M. Arlen, Massimo Fantini

**Affiliations:** Precision Biologics, Inc., 4922 Fairmont Ave., Suite 320, Bethesda, MD 20814, USA; anjum.zaki@precision-biologics.com (A.Z.); sharon.mavroukakis@precision-biologics.com (S.A.M.); philip.arlen@precision-biologics.com (P.M.A.); massimo.fantini@precision-biologics.com (M.F.)

**Keywords:** monoclonal antibody, affinity maturation, O-glycans, internalization, tumor specificity, ADC

## Abstract

**Background:** Enhancing the binding affinity of monoclonal antibodies (mAbs) has the potential to improve their therapeutic efficacy. In this study, we generated a novel mAb, PB-223, through affinity maturation of the parental antibody NEO-102. NEO-102 (Ensituximab) is a chimeric human IgG1 mAb that targets a cancer-specific glycosylated variant of MUC5AC expressed on colorectal and pancreatic cancers while sparing healthy tissues. In a Phase 2 clinical study involving heavily pretreated patients with advanced, refractory colorectal cancer, NEO-102, as a monotherapy, exhibited modest efficacy. **Methods:** We engineered the VH and VL regions of NEO-102 through affinity maturation to enhance antigen binding while preserving target specificity. The optimized clone, PB-223, was evaluated for improved binding by Biacore T200, flow cytometry, and immunohistochemistry (IHC). The specific PB-223 target antigen was discovered using an O-glycan array. An internalization assay evaluated the ability of PB-223 to be internalized into human cancer cell lines expressing its target antigen. **Results:** Analysis performed with the Biacore T200 evaluation (version 3.2) software revealed that PB-223 exhibits a 4.55-fold lower equilibrium dissociation constant (KD) compared with NEO-102 towards recombinant human Bovine Submaxillary Mucin (BSM), a protein rich in O-glycans. PB-223 has enhanced binding to human cancer cell lines recognized by NEO-102 using flow cytometry. O-glycan array analysis identified O-glycans terminating with α(2,6) sialic acids, including core 2 O-glycans and sTn glycans, expressed by human cancer cell lines reactive with PB-223 in flow cytometry, as the specific binding epitope of PB-223. IHC analysis of human tumor tissues showed that PB-223 does not bind to healthy tissues tested in this study, and that demonstrates an increase in the number of cancer tissues recognized and in the IHC score compared to NEO-102. PB-223 is internalized into a human cancer cell line expressing its target antigen. **Conclusions:** PB-223 can potentially be used as a targeting moiety for antibody-based therapeutics, including antibody–drug conjugates (ADCs), bispecific antibodies, immune-engaging constructs, and radiopharmaceuticals, for the treatment of human cancers expressing O-glycans terminating with α(2,6) sialic acids, including core 2 O-glycans and sTn glycans.

## Simple Summary

Strategies to improve the efficacy and safety of monoclonal antibodies (mAbs) as anticancer drugs include enhancing their affinity for their target antigen and developing mAbs with high specificity for targets expressed by cancer cells only. In this study we have generated a new mAb, named PB-223, by enhancing the affinity of the mAb NEO-102 for O-glycans attached to proteins, such as MUC5AC, specifically expressed by human cancers but not by healthy tissues. We showed that PB-223 binds to O-glycans terminating with α(2,6) sialic acids, including core 2 O-glycans and sTn glycans, expressed specifically by two human cancer cell lines tested in this study and that binds to a wide variety of human cancer tissues. PB-223 does not bind to healthy human tissues or to most normal human tissues adjacent to malignant tumors tested in this study. Furthermore, PB-223 can be internalized into a human cancer cell line expressing its target antigen. These findings provide a strong rationale to use PB-223 to build antibody-based therapeutics.

## 1. Introduction

Affinity maturation is a critical strategy in therapeutic antibody development, enabling optimization of antigen binding to improve clinical efficacy of chimeric, humanized, and fully human monoclonal antibodies (mAbs) [1,2]. The enhanced binding affinity of therapeutic antibodies for their targets can translate into meaningful clinical advantages, including lower effective dosing, improved target engagement, reduced off-target toxicity, and decreased treatment costs. As a result, affinity optimization has become a standard component of modern antibody engineering pipelines [1,2,3,4].

Physiologically, affinity maturation of antibodies occurs in germinal centers, where antigen-experienced B cells undergo multiple rounds of somatic hypermutation and selection, yielding antibodies with progressively improved affinity, avidity, and functional activity for their target antigen [5,6]. In vitro affinity maturation recapitulates this process to generate mAbs with superior target engagement. The main steps of in vitro affinity maturation include increasing the diversification of the antibody base sequence, followed by rigorous selection of higher affinity binders and by validation of individual clones for their affinity to the target antigen [1,5,7].

Multiple in vitro strategies have been successfully applied to perform affinity maturation of mAbs, including random or targeted mutagenesis, chain shuffling and in silico approaches. The first two methods involve the employment of display technologies (i.e., phage, bacterial, yeast, and mammalian cell display), while the in silico methodology requires computer-aided design [1,4,8]. Clinically effective therapeutic mAbs generally exhibit binding affinities in the low-nanomolar or sub-nanomolar range (≤1 nM), and many marketed mAbs have undergone multiple rounds of affinity maturation to reach this threshold, with reported improvements of up to 100-fold [7,8,9,10].

In the present study, we sought to improve the therapeutic potential of NEO-102 (Ensituximab) through affinity maturation. NEO-102 is a chimeric human IgG1 mAb that recognizes a cancer-specific glycosylated variant of MUC5AC selectively expressed in colorectal and pancreatic cancers, with minimal expression in normal tissues [11]. NEO-102 was originally derived from mice immunized with the Hollinshead allogeneic colorectal cancer vaccine platform, which contains immunogenic tumor-associated antigens (TAAs) isolated from membrane fractions derived from surgically resected human colorectal cancers [11,12,13]. Following immunization, murine mAbs were screened for their reactivity against TAAs contained in the vaccine and against several human colon and pancreatic cancer cell lines. One of the most reactive murine mAbs was purified and named NPC-1. Then, murine variable regions of NPC-1 were fused in-frame with human heavy-chain (HC) and light-chain (LC) IgG1 constant regions to create the chimeric antibody NPC-1C (NEO-102) [11]. Functionally, NEO-102 mediates tumor cell killing primarily through antibody-dependent cellular cytotoxicity (ADCC) [11].

In a phase II clinical trial involving patients with advanced, refractory colorectal cancer, NEO-102 demonstrated a favorable safety profile but only modest antitumor activity as a monotherapy, with stable disease observed in 21% of evaluable patients [14]. These clinical findings suggested that while target selection and safety were appropriate, further optimization of NEO-102’s functional properties might be required to enhance its therapeutic efficacy.

One plausible contributor to the limited clinical activity of NEO-102 is its moderate binding affinity for the target antigen. The dissociation constant (KD) of NEO-102 was measured at 5.60 × 10^−9^ M [15], a value that, while within the nanomolar range, may be suboptimal for sustained target engagement in the tumor microenvironment (TME). 

The primary objective of this study was to enhance the binding affinity of NEO-102 using Fast Screening for Expression, Biophysical Properties, and Affinity (FASEBA), with the goal of improving tumor targeting and downstream effector function.

FASEBA is a rapid, high-throughput platform that enables early-stage selection of antibody variants with improved affinity, expression, and biophysical stability. FASEBA uses an Escherichia coli-based system, without the need for protein purification. From a translational perspective, FASEBA allows for efficient identification of leading candidates in a short amount of time, providing information on expression levels, biophysical properties, and affinities at an early stage of protein engineering. This allows for a reduction in risk during downstream manufacturing and clinical development [16]. In this study, using FASEBA, we engineered the variable heavy-chain (VH) and light-chain (VL) domains of NEO-102 and identified a novel antibody clone, AHF18095 (PB-223), with enhanced binding to the target antigen.

We also reported comparative analyses of PB-223 and NEO-102 using flow cytometry and IHC across multiple tumor cell lines and human tumor tissues to assess improvements in antigen recognition. In addition, an O-glycan microarray was employed to define the O-glycan epitope recognized by PB 223, providing mechanistic insight into its tumor specificity. Finally, antibody internalization studies were performed on the human ovarian cancer cell line OV-90 expressing the PB-223 target antigen, supporting the translational relevance of PB-223 for potential therapeutic applications for the treatment of solid tumors expressing its target antigen.

## 2. Materials and Methods

### 2.1. Cell Lines and Culture

The following human carcinoma cell lines were obtained from the American Type Culture Collection (ATCC) (Manassas, VA, USA): OV-90 (ovarian cancer), SW-480, SW-403, LoVo, COLO 205 (colorectal cancer), DU 145, PC-3 (prostate cancer), SK-BR-3 (breast cancer), HCC1937 (triple negative breast cancer). All human cancer cell lines were maintained in a culture medium (Corning Life Science, Manassas, VA, USA) designated by the provider for propagation and maintenance. The culture medium was supplemented with 10% USA-sourced and heat-inactivated HyClone fetal bovine serum (GE Healthcare Life Sciences, Issaquah, WA, USA), 100 U/mL penicillin and 100 μg/mL streptomycin (Corning Life Science, Manassas, VA, USA).

### 2.2. Antibody Affinity Maturation

The chimeric IgG1 mAb NEO-102 was generated by Precision Biologics from the Hollinshead vaccine platform, as previously described [11].

The binding affinity of NEO-102 for its target antigen was enhanced using the FASEBA screening platform and was performed by ProBio [16].

The affinity maturation consisted of several steps:

1. Construction and production of NEO-102 Fab FASEBA sample: The DNA sequences encoding the antibody heavy and light chains were synthesized and inserted into the FASEBA vector to construct expression plasmids for the Fab parent. Then the FASEBA vector was transferred into TG1-competent cells, and after selecting positive clones for culture, Isopropil-β-D-1-tiogalattopiranoside (IPTG) was used to induce Fab parental antibody expression.
2. Enzyme-linked immunosorbent assay (ELISA) validation of NEO-102 Fab FASEBA sample: Recombinant human Bovine Submaxillary Mucin (BSM) (Sigma-Aldrich, St Louis, MO, USA), a protein rich in O-glycans, was used as the target antigen for NEO-102 binding. In total, 100 µL of microplate coating solution, containing BSM 10 µg/mL dissolved in 1× phosphate-buffered saline (PBS) pH 7.2, was added to each well in a 96-well plate, and the plate was incubated at 4 °C overnight. The plate was then washed with 0.05% PBST (1× PBS + 0.05%Tween-20) and then incubated with FASEBA supernatant at 37 °C for 1 h. Subsequently, the plate was incubated with MPBS (3% Nonfat-Dried Milk + 1× PBS) at 37 °C for 1 h. Then BSM-biotin was added in a two-fold serial dilution (from 2 µg/mL to 0.0078 µg/mL) at room temperature (RT) for 2 h. Then, the plate was incubated at RT for 45 min with 0.001 μg/mL streptavidin–HRP, dissolved in 0.05% PBST, to detect the BSM-biotin. The plate was then washed with 0.05% PBST, and the absorbance was measured at 450 nm following GenScript’s standard operating procedure (SOP). Antigens with an OD450 ranging from 0.5 to 0.8 were selected for subsequent FASEBA ELISA screening.
3. Construction of precise mutagenesis library (PML): In total, 57 residues in CDR regions were mutated into the 19 desired amino acids using optimal codons for *E. coli*. DNA oligonucleotide library synthesis was performed on a programmable microarray. The Saturation Scanning Mutagenesis library was synthesized through advanced GenScript oligonucleotide techniques and cloned into the U6901GJ130-Fab-Parental-pFASEBA vector as a sub-pool. Each individual PML library was generated per residue based on the FASEBA platform with a theoretical diversity of 20. The library quality was ensured through next-generation sequencing (NGS) with a minimum coverage of 90%. In total, 44–48 clones were randomly selected from each PML for expression in *E. coli*.
4. FASEBA screening and affinity ranking: Individual 44–48 PML library clones were inoculated and induced for expression in 96-deep-well plates. The crude protein secreted in the medium was analyzed by ELISA against BSA and BSM protein for the assessment of expression and binding affinity compared with parental FASEBA supernatant, NC (non-related FASEBA supernatant), and a blank (2YT medium). Clones with improved value were selected for sequencing. The “beneficial mutants” were confirmed by affinity ranking. Off-rate screening was performed on a Biacore T200 (version 3.2). The selected clones of the Fab single-domain antibody against serum albumin (Fab-SASA) were secreted into the culture medium and captured by SASA capture biosensors. After equilibration, the antigen was injected for 120 s (association phase), followed by the injection of running buffer HBS-EP (10 mM HEPES, 500 mM NaCl, 3 mM EDTA, 0.05% Tween 20, pH 7.4) for 360 s (dissociation phase). The surface was regenerated before the injection of the other selected clones. The process was repeated until all samples were analyzed. The off-rates of Fab-SASA clones were obtained by fitting the experimental data locally to a 1:1 interaction model using the Biacore T200 evaluation (version 3.2) software. The selected mutants were ranked by their dissociation rate constants (off-rates, kd).
5. Construction and screening of combinatorial library: Once the “beneficial mutants” were identified, a combinatorial library was constructed with random combinations of these mutations by Polymerase Chain Reaction (PCR). The combinatorial clones were analyzed by ELISA and selected for DNA sequencing and affinity ranking. The top combinations of “beneficial mutants” with the highest affinity increase, without compromising expression, were finally selected for antibody affinity measurement.
6. IgG construction, expression and purification: The variable domains of heavy-chain- and light-chain-encoding affinity-matured antibodies were synthesized and inserted into the pCDNA3.4 vector to construct full-length IgG-expressing vectors. The heavy- and light-chain-expressing plasmids were used for transfection. The recombinant IgGs secreted into the medium were purified using protein-A affinity chromatography. The concentration and purity of proteins were assessed by OD280 and SDS-PAGE, respectively.
7. Affinity measurement of purified affinity-matured antibodies: The affinity of purified antibodies binding to the antigen was individually determined using Biacore T200. Antibodies were captured on the sensor chip. The target antigen was used as the analyte. The data for dissociation (kd) and association (ka) rate constants were obtained using the Biacore evaluation software (version 3.2). The equilibrium KD was calculated from the ratio of kd over ka.

### 2.3. Identification of Binding Epitope of PB-223

To investigate if PB-223 recognizes O-glycans expressed on proteins from cancer cells, we tested the binding of PB-223 to an O-glycan array consisting of 94 O-glycans. O-glycan array was obtained from Creative Proteomics (Shirley, NY, USA).

The array structures of the 94 O-glycans were already reported in a previous study [17].

The microarray was first treated with Glycan Array Blocking Buffer (GABB) at RT for 30 min. Subsequently, PB-223 was diluted in Glycan Array Assay Buffer (GAAB) at various concentrations (20, 10, 2 µg/mL) and then applied to the microarray, followed by incubation at RT for 1 h. After the incubation period, the microarray was thoroughly washed, and anti-human IgG Fc (Cy3) was added to the microarray at a concentration of 5 µg/mL for 1 h at RT. After incubation, the microarray was washed again and then subjected to scanning.

The microarray was scanned at 532 nm using an Innopsys InnoScan 710 Microarray Scanner (Innopsys, Carbonne, France) with a high-power laser intensity (1 PMT). Microarray data were analyzed using the Mapix software (Innopsys). The software was used to detect each spot on the array and calculate the relative fluorescence unit (RFU) intensity for each spot. Background RFU was subtracted from each spot’s RFU value. The details of this procedure are described in a previously published article [17].

### 2.4. Flow Cytometry

The binding affinity between NEO-102 and the affinity-maturation-generated clone AHF18095 (PB-223) was evaluated by flow cytometry.

Human cancer cell lines were harvested, washed twice with 1 mL of 1× PBS no Ca++, no Mg++ (Thermo Fisher Scientific, Waltham, MA, USA), and then incubated with 1 µL per test of LIVE/DEAD Fixable Near-IR (Thermo Fisher Scientific, Waltham, MA, USA) in 1 mL 1× PBS for 20 min in the dark at RT to accomplish live-versus-dead-cell discrimination. After the incubation, cells were washed once with 1 mL 1× PBS, followed by a second wash with 1 mL of pre-chilled FACS buffer (1× PBS + 1% BSA).

To evaluate the reactivity of cancer cells with NEO-102 and PB-223, the cells were then incubated for 30 min at 4 °C in 100 µL of FACS buffer containing 20 µg/mL unconjugated NEO-102 or PB-223. After incubation, cells were washed twice with 2 mL of FACS buffer and then incubated for 30 min at 4 °C in 100 µL of FACS buffer containing 20 µg/mL of mouse anti-human IgG1 Fc secondary antibody AF488 (Thermo Fisher Scientific, Waltham, MA, USA). After incubation, cells were washed twice with 2 mL of FACS buffer and examined using a BD LSRFortessa flow cytometer (BD Biosciences, San Jose, CA, USA). The analysis of cellular fluorescence was performed using BD FACSDiva Software (version 8.0) (BD Biosciences, San Jose, CA, USA). Positivity was determined by comparing unstained cells with cells stained with NEO-102 or PB-223. The percentage of PB-223 or NEO-102 positive cells was calculated with the following formula: % PB-223 or NEO-102 stained cells − % unstained cells. Cells with staining values > 10% were considered positive. 

### 2.5. O-Glycan Profiling of Cell Lines Reactive with PB-223 

OV-90, HCC1937 and LoVo cell lines were profiled for the expression of O-glycans.

O-glycan profile analysis was performed by Creative Proteomics (Shirley, NY, USA) using a procedure previously described [17]. The profiling procedure involved several steps, encompassing N-glycans removal, O-glycan preparation, permethylation and MS MALDI analysis.

### 2.6. IHC on Human Malignant Tumor Tissues and Normal Tissues Adjacent to the Tumor

IHC analysis of reactivity of PB-223 and NEO-102 against human malignant tumor tissues and normal tissues adjacent to the tumor was performed by Boster Bio (Pleasanton, CA, USA). Human malignant tumor tissues and normal tissues adjacent to the tumor were derived from human tissue microarray (TMA) BCN601a (https://www.tissuearray.com/tissue-arrays/Multiple_Organ_Tumor/BCN601a, accessed on 8 November 2025), BCN721b (https://www.tissuearray.com/tissue-arrays/Multiple_Organ_Tumor/BCN721b, accessed on 8 November 2025) and BR1102 (https://www.tissuearray.com/tissue-arrays/Breast/BR1102, accessed on 8 November 2025).

BCN601a contains 8 cases of cancer for each of the following organs: prostate, pancreas, colon, kidney, lung, and uterus. BCN721b contains multiple cases of cancer with normal tissue adjacent to the tumor for each of the following organs: esophagus, stomach, colon, rectum, liver, lung, kidney, breast, cervix, ovary, prostate and pancreas. BR1102 contains multiple cases of breast cancer, including triple-negative breast cancer, with normal tissue adjacent to the tumor. Cancer tissues were run and analyzed using the Leica Bond Max Automated Immunohistochemical Staining Procedure (Leica Biosystems, Buffalo Grove, IL, USA). The IHC staining procedure was performed using the Human-on-Human HRP-Polymer kit (Biocare Medical, Pacheco, CA, USA). PB-223 and NEO-102 were used as the primary antibodies. PB-223 and NEO-102 were first tagged with digoxigenin, combining them with a digoxigenin anti-human linker, to detect them on human tissue with an HRP-polymer system. Then digoxigenin-tagged PB-223 and NEO-102 were used as primary antibodies at 1 µg/mL for 1 h at RT. Tissues were then washed with washing buffer, and a mouse anti-digoxigenin secondary antibody (BRR4055G; Biocare Medical, Pacheco, CA, USA) was used as the secondary antibody for 30 min at RT. Then the tissues were washed and incubated with MACH 2 Mouse HRP-Polymer (MHRP520G; Biocare Medical, Pacheco, CA, USA) for 30 min at RT. Then the tissues were washed and incubated with DAB and hematoxylin and analyzed with Leica Bond Max (Leica Biosystems, Buffalo Grove, IL, USA).

The percentage of tumor cells in cancer tissues and normal tissues adjacent to the tumor that react with PB-223 and NEO-102 and the relative staining intensity (IHC score) were determined by a review of stained tissues from a single board-certified pathologist blinded to antibody identity. IHC score was determined using a semi-quantitative scale: score 1, weak staining; score 2, moderate staining; and score 3, strong staining.

Tissues with an IHC score of 0 were considered negative for the expression of the antigen recognized by NEO-102 and PB-223. Scores were assigned based on the intensity and extent of membranous/cytoplasmic staining observed in the tissue sections and the extent of positive cells within each tissue section. In this study, colon carcinoma tissues with an IHC score of 3 and 100% positive cells were used as positive controls for NEO-102 and PB-223 staining.

The details of the IHC staining procedure are reported in the Supplementary Materials in the Methods Section.

### 2.7. IHC on Normal Human Tissues

Immunochemistry analysis was performed using normal human tissues from the brain, liver, lung, colon, and lymph nodes and the positive control human cancer cell line OV-90 fixed in buffer for FFPE and sectioning. FFPE sectioning was performed by iHisto Inc, (Salem, MA, USA) using a Fisherbrand Superfrost Plus slide. These normal tissues encompassed a diverse range of normal tissues across multiple organs. Specifically, the samples included 1 case of normal human brain and 3 cases each of normal human liver, lung, colon, and lymph node. Rat tissue was used as the control.

The FFPE slides were deparaffinized and rehydrated through the following steps: two 15 min immersions in xylene, followed by two 5 min immersions in 100% alcohol and a 5 min immersion in 70% alcohol. Then, the slides were removed from 70% alcohol and washed in water. Then, antigen retrieval buffer (citric acid-based; pH 6) was added to the slides, and antigen retrieval was completed by putting the slides in a pressure cooker at 110 °C for 15 min. Slides were then allowed to cool down for 20 min, and then they were brought to RT by slowly adding water to the retrieval container. 

The slides were then washed twice for 5 min in TBS (Tris-buffer saline), followed by a final wash with TBST (Tris-buffer saline with Tween 20). Then, a peroxidase blocking solution was applied to the slides at RT for 10 min. Slides were then washed again with TBS and TBST, as described above. Slides were then blocked with 2.5% normal horse serum and incubated at RT for 30 min. After blocking, slides were incubated overnight at 4 °C with PB-223 (10 µg/mL) or a mouse IgG1 isotype control antibody (10 µg/mL) diluted at a ratio of 1:150 in normal horse serum. Both PB-223 and the isotype control had the murine Fab region. 

The following morning, the slides were washed again with TBS and TBST, as previously described. Then, the slides were incubated with the secondary antibody (horse anti-mouse HRP directed against the murine Fab region of the primary antibody PB-223 or isotype control) for 1 h at RT. The slides were washed again with TBS and TBST; then, DAB was applied to each section, and the stain was allowed to develop for 10 min. Finally, the sections were counterstained with hematoxylin and coverslipped. Detection was carried out using a Pannoramic^®^ 1000 Scanner (3DHISTECH, Budapest, Hungary) for bright-field scanning.

### 2.8. Internalization Assay

A live cell image-based internalization assay (Incucyte assay) was employed to observe the internalization process of PB-223 within the OV-90 cell line. As a validation measure, the interaction between the mAb Herceptin and its target human breast cancer cell line SK-BR-3 was used as the system control. The internalization assay was performed by ProBio.

For the internalization assay, cells were first digested with Accutase cell digestion solution (natural enzyme mixture with proteolytic and collagenolytic enzyme activity), harvested by centrifugation, and then resuspended in assay buffer (complete culture medium).

After adjusting the target cell density, the target cell suspension was transferred to the assay plate according to the map schemes. The assay plate was then incubated in a cell incubator (37 °C with 5% CO_2_) overnight.

The following day, target cells were incubated with working solutions containing Herceptin (Roche, Basilea, Switzerland), PB-223 and an Anti-HEL Human IgG1 isotype control (Abinvivo, Metuchen, NJ, USA) at 37 °C with 5% CO_2_ for 48 h to allow for binding and internalization. Herceptin was added in a two-fold serial dilution from 30 nM to 0.23 nM; PB-223 and the IgG1 negative control were added in a two-fold serial dilution from 60 nM to 0.47 nM. After incubation, images depicting internalization were captured using the Incucyte^®^ Live-Cell Analysis System with an appropriate fluorescent module configured for the indicated time. Raw data and results were analyzed and exported with the Incucyte^®^ Live-Cell Analysis System (Incucyte, Gottingen, Germany).

The Incucyte^®^ Human FabFluor-pH Red Antibody Labeling Dye (Sartorius, New Oxford, PA, USA) was used to perform, in real time, the kinetic evaluation of Herceptin and PB-223 internalization into their target cells. This dye is tailored to rapidly label Fc-containing antibodies with a Fab-fragment-conjugated pH-sensitive fluorophore. The pH-sensitive dye-based system capitalizes on the acidic environment of the lysosomes to quantify internalization of the labeled antibody. In this process, Fabfluor-labeled antibodies initially reside in the neutral extracellular solution (pH 7.4), where they interact with cell-surface-specific antigens and undergo internalization. Upon entry into the lysosomes, characterized by an acidic environment (pH 4.5 to 5.5), there is a noticeable increase in fluorescence. In the absence of the expression of the specific antigen, internalization does not occur, as determined by the low fluorescence intensity of the labeled antibodies. Incucyte-integrated analysis software (v2020c) was used to minimize background fluorescence.

### 2.9. Statistical Analysis

Data were analyzed using the GraphPad Prism 10.0.2 software (GraphPad Software, La Jolla, CA, USA). Comparisons between the two groups were conducted by an unpaired *t*-test. Differences were considered significant when the *p* value was <0.05.

## 3. Results

### 3.1. Generation of PB-223 by Enhancing the Binding Affinity of NEO-102 for BSM Through FASEBA Screening Platform

The binding affinity of NEO-102 for its target antigen was enhanced by engineering the VH and VL sequences of NEO-102, according to the strategy of PML saturation mutagenesis and FASEBA screening, with the aim of maintaining binding to the target antigen while achieving a lower KD. As the first step, the DNA sequences encoding NEO-102 heavy and light chains were synthesized and inserted into the FASEBA vector to construct expression plasmids of the Fab parent. Fab parental antibody expression was induced from the FASEBA vector using IPTG, as described in the Section 2. Fab FASEBA samples were then validated by ELISA using recombinant BSM at different concentrations as the target antigen. A concentration of 0.0078 µg/mL for the BSM-biotin protein was selected for further PML library screening. For the PML library construction, each individual PML library was generated per residue based on the FASEBA platform with a theoretical diversity of 20 (Table 1). Library quality was ensured through NGS with a minimal coverage of 90%.

From each PML library, more than 44 clones were randomly selected for sequencing. A total of 2624 individual clones were tested for binding activity by ELISA, compared with the parental FASEBA supernatant, NC (non-related FASEBA supernatant), and a blank (2YT medium).

Out of the 2624 clones tested, only 36 showed increased affinity compared to the parental supernatant. These clones were sent for DNA sequencing. From the clones analyzed, eight residues showed improved binding affinity. These residues were considered “beneficial mutants”. Aminoacidic mutations in specific VL and VH regions are shown in Table 2.

Once the “beneficial mutants” were identified, a combinatorial library was constructed with random combinations of these mutations by PCR. From the combinatorial library, 276 clones were randomly selected for binding with BSM in ELISA, compared with parental and NC samples. The clones of each plate with increased OD compared to the parental clone (OD ratio between mutant clones and parental clone >1.5) were sent for DNA sequencing. A total of 15 clones were sequenced. The OD ratio and the specific aminoacidic mutation in the VH and VL regions of these 15 clones compared to the parental clone are shown in Table 3.

The 15 clones were then sent for affinity ranking. The affinity of BSM to the supernatant of the selected clones was compared to NEO-102 using the Biacore T200 evaluation software (version 3.2). The data for the dissociation (kd) and association (ka) rate constants and the equilibrium KD are reported in Table 4.

The three clones (AHF18095, AHF18100, and AHF18104) with the lowest KD and the highest increase in the KD ratio compared to the wild-type clone (NEO-102) were selected for antibody production and purification. As shown in Table 4, the clone AHF18095 exhibited the highest increase in the KD ratio, compared to NEO-102, towards BSM (4.55-fold improvement in binding affinity). For this reason, the clone AHF18095 was then selected for further experiments and was designated PB-223.

### 3.2. PB-223 Binds to O-Glycans Terminating with α(2,6) Sialic Acids, Including Core 2 O-Glycans and sTn Glycans

We previously demonstrated that NEO-102 targets a glycosylated variant of MUC5AC specifically expressed by colorectal and pancreatic cancers but not by healthy tissues [11]. Glycosylation is a post-translational modification that occurs in mammalian cells and is often disrupted in cancer cells. One of the most disrupted glycosylation patterns during carcinogenesis is the synthesis of O-glycans attached to the proteins of cancer cells. The presence of incomplete/truncated O-glycans attached to the proteins of cancer cells is associated with tumor progression, metastasis and poor prognosis [18,19].

To evaluate if PB-223 can bind to O-glycans, in this study, we performed an O-glycan array to test the binding activity of PB-223 to 94 different O-glycan structures.

**Figure 1:**
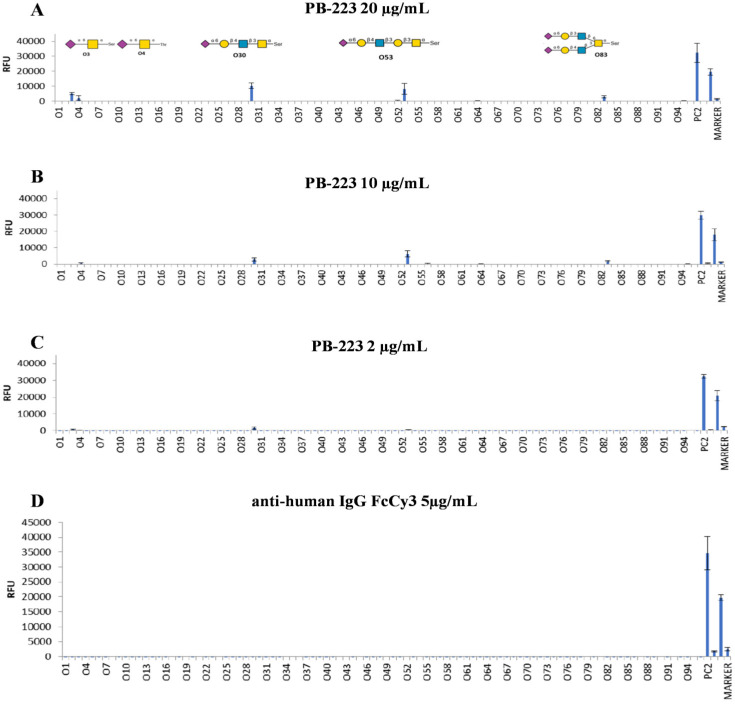
Analysis of PB-223 binding to the O-glycan array. Analysis of PB-223 binding to the O-glycan array. (**A**–**C**) PB-223 was used at concentrations of 20, 10 and 2 µg/mL. (**D**) Anti-human IgG FcCy3 was used at 5 µg/mL. The microarray was scanned at 532 nm using an Innopsys InnoScan 710 Microarray Scanner (Innopsys, Carbonne, France) with a high-power laser intensity (1 PMT). Subsequently, Innopsys’s Mapix software was employed to further analyze the array scan. The software was used to detect each spot on the array and calculate the relative fluorescence unit (RFU) intensity for each spot. Background RFU was subtracted from each spot’s RFU value. RFU scale is presented on the vertical axes. The binding signals were determined by subtracting background signals and signals from negative control spots. PC2: human IgG; marker: streptavidin Cy3 and Cy5.

As shown in Figure 1, the microarray data revealed that PB-223 selectively binds to O-glycans terminating with α(2,6) sialic acids in a dose-dependent manner. These include both sTn antigens (O3 and O4) and core 2 O-glycan structures (O30, O53 and O83).

It is important to note that PB-223 showed the strongest binding to core 2 O-glycan configurations containing a Neu5Ac(α2-6) Gal (β1-4) GlcNAc epitope in the β1-3 linkage (O30 in its free form and O53 with β1-3 linkage), while the epitope present in the biantennary glycoform (O83) diminishes the ability of the antibody to recognize the antigen (Figure 1A). Array analysis disclosed that anti-human IgG Fc (Cy3) alone does not interact with glycans on the O-glycan array (Figure 1D).

### 3.3. PB-223 Shows a Stronger Binding than NEO-102 to Human Cancer Cell Lines Expressing Core 2 O-Glycans

To further confirm that O-glycans terminate with α(2,6) sialic acids, including the core 2 O-glycans and sTn glycans, are the real target of PB-223, we profiled different human carcinoma cell lines for PB-223 binding using flow cytometry, and we compared the percentage of cells reactive with PB-223 vs. NEO-102.

As reported in Table 5, SW-403, COLO 205, HCC1937, and OV-90 cell lines were considered positive for binding with PB-223 and showed a strong reactivity with PB-223, while LoVo, SW-480, and DU 145 were negative. The PC-3 cell line showed modest reactivity with PB-223. In addition, in all cell lines considered positive for binding with PB-223, we observed a more than 2-fold increase in the percentage of positive cells compared with NEO-102 [SW-403 (2.22-fold increase), COLO 205 (3.96-fold increase), HCC1937 (2.69-fold increase), and OV-90 (2.15-fold increase)], although the only statistically significant difference in the percentage of positive cells reactive with PB-223 compared to NEO-102 was observed in the OV-90 cell line (Table 5). The absence of a statistically significant difference in the percentage of positive cells reactive with PB-223 compared to NEO-102 in SW-403, COLO-205 and HCC1937 is due to the high standard deviation between the experiments. Binding curves comparing the reactivity of NEO-102 and PB-223 for each cell line tested in this study, in each experiment, are reported in Supplemental Figure S1.

To further prove that cell lines reactive with PB-223 express O-glycans recognized by PB-223, two cell lines strongly reactive with PB-223 (HCC1937 and OV-90) and one non-reactive cell line with PB-223 in flow cytometry (LoVo) were screened for the expression of O-glycans. As reported in Table 5, the most abundant O-glycan recognized by PB-223 expressed by the HCC1937 and OV-90 cell lines is the Neu5Ac(α2-6) Gal (β1-4) GlcNAc epitope with the β1-3 linkage (O53; core 2 O-glycan). The expression of this glycan on the LoVo cell line was very limited (0.29%).

The profile of O-glycans expressed by these cell lines, including monoisotopic mass, proposed compositions, proposed structures, and the relative abundance, is reported in Supplemental Figure S2. 

### 3.4. PB-223 Demonstrates an Increase in the Number of Cancer Tissues Recognized and in the IHC Score Compared to NEO-102 by IHC 

To further confirm data obtained in flow cytometry using human cancer cell lines, PB-223 and NEO-102 reactivity against human malignant tumor tissues was evaluated by IHC.

TMAs containing prostate, pancreas, colon, rectum, kidney, lung, esophagus, stomach, liver, breast, uterus, cervix, and ovary tumor tissues were profiled for reactivity with both NEO-102 and PB-223.

As shown in Table 6, both PB-223 and NEO-102 showed similar reactivity against pancreas adenocarcinoma (9/10 tissues), rectum adenocarcinoma (3/3 tissues), lung adenocarcinoma (3/4 tissues), endometrioid adenocarcinoma (4/8 tissues), cervix squamous cell carcinoma (1/3 tissues), and high-grade serous carcinoma of the ovary (2/3 tissues).

**Figure 2:**
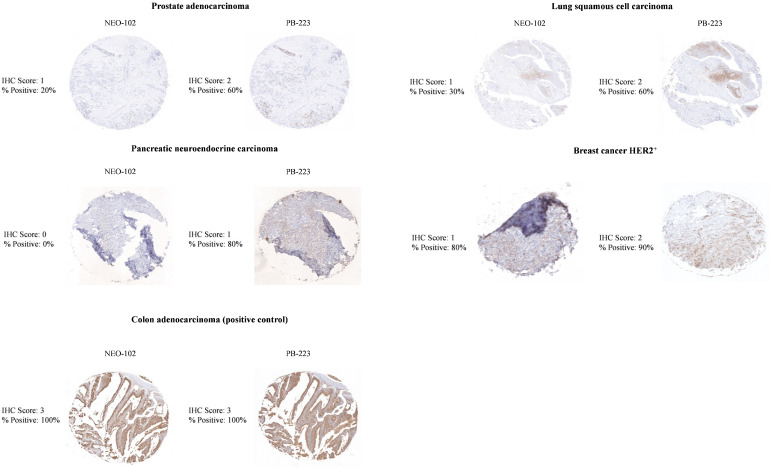
IHC staining of human malignant tumor microarray tissues by PB-223 compared to NEO-102. The figure depicts representative human malignant tumor microarray tissues from the prostate, pancreas, lungs and breasts, as well as a positive control (colon) stained with PB-223 compared to NEO-102. All images were obtained at 20× magnification. All specimens were imaged under identical settings.

PB-223 demonstrated increase in the number of tissues recognized and in the IHC score compared to NEO-102 in prostate adenocarcinoma (3/11 tissues vs. 2/11 tissues) and colon adenocarcinoma (10/11 tissues vs. 7/11 tissues), although this increase was not statistically significant. It is important to note that PB-223 was able to recognize one pancreas neuroendocrine carcinoma that was not reactive with NEO-102 (Figure 2, Table 6). Although we did not observe a difference in the number of tissues recognized by PB-223 or NEO-102 in lung squamous cell carcinoma, HER2+ breast cancer, or triple-negative breast cancer, tissues reactive with PB-223 showed a higher IHC score compared to tissues reactive with NEO-102 (Figure 2, Table 6), but the increase in IHC score was not statistically significant. No reactivity with PB-223 or NEO-102 was detected in kidney clear cell carcinoma, esophageal squamous cell carcinoma, stomach adenocarcinoma, or hepatocellular carcinoma (Table 6).

### 3.5. PB-223 Does Not React by IHC to Normal Human Tissues from Brain, Liver, Lungs, Colon, or Lymph Nodes or to Most Normal Human Tissues Adjacent to Malignant Tumors

To evaluate whether PB-223 retained the high specificity of NEO-102 to bind only to malignant tumor tissues, we compared the reactivity of PB-223 and NEO-102 against normal human tissues and normal human tissues adjacent to malignant tumors using IHC.

Normal human tissues adjacent to malignant tumors are derived from the same TMAs containing malignant tumors described in the previous paragraph.

**Figure 3:**
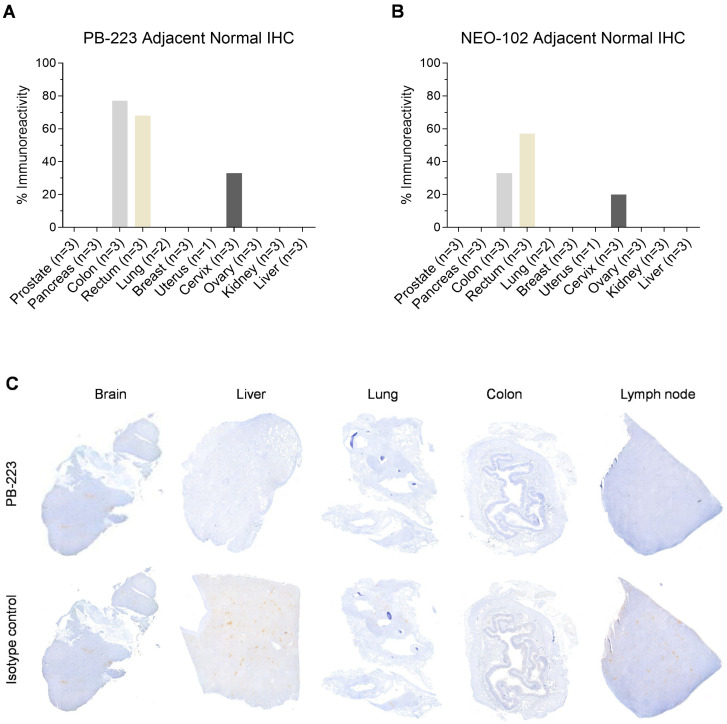
IHC staining of human normal tissues and normal tissues adjacent to tumors by NEO-102 and PB-223. (**A**). Quantification of PB-223 positive staining of normal human tissues adjacent to malignant tumors derived from TMA samples. (**B**). Quantification of NEO-102 positive staining of normal human tissues adjacent to malignant tumors derived from TMA samples. (**C**). Representative PB-223 staining of normal brain, liver, lung, colon, and lymph node tissues compared to isotype control. n: number of samples.

As shown in Figure 3A,B, immunoreactivity with both PB-223 and NEO-102 was completely absent from normal human tissues adjacent to malignant prostate, pancreas, lung, breast, uterus, ovary, kidney, and liver tumors.

We observed reactivity with PB-223 and NEO-102 in 3/3 normal human tissues adjacent to malignant colon and rectum tumors (reactivity with goblet cells) and in 1/3 of normal human tissues adjacent to a malignant cervical tumor (reactivity with endocervical glandular cells).

IHC staining of normal tissues demonstrated that PB-223 does not bind to normal human brain, liver, lung, colon, and lymph node tissues as compared to the isotype control (Figure 3C).

Altogether, these data indicate that PB-223 can bind specifically to human malignant tumor tissues from a wide variety of carcinomas, can recognize additional human malignant tumor tissues compared to NEO-102, and it does not bind to human normal tissues or most normal human tissues adjacent to malignant tumors.

### 3.6. PB-223 Is Internalized into a Human Cancer Cell Line Expressing Its Target Antigen

The ability of mAbs to be internalized into cancer cells after binding with their target antigens is a crucial feature of using mAbs as tools to specifically deliver cytotoxic drugs into cancer cells. This is the case for ADCs, where mAbs are engineered to deliver potent anticancer drugs specifically into cancer cells after internalization [20].

To evaluate PB-223’s ability to be internalized into human cancer cells expressing its target antigen, we performed an internalization assay using the OV-90 cancer cell line as the target cell.

As a positive control for the assay (system control), we used Herceptin as a mAb and the human breast cancer cell line SK-BR-3 as the target cells.

**Figure 4:**
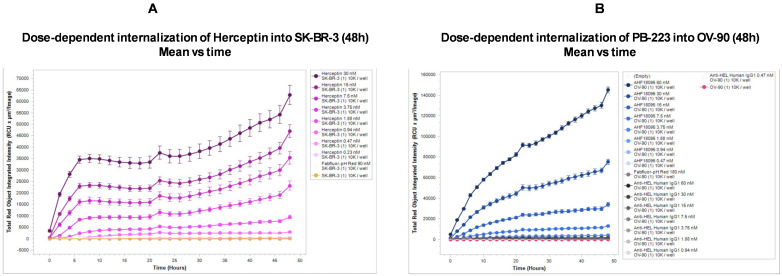
In vitro internalization of PB-223 (AHF18095) into OV-90 cancer cell line. The figure shows dose-dependent internalization of Herceptin and PB-223. (**A**) SK-BR-3 cancer cell line was treated with the system control (Herceptin). Herceptin was added in a two-fold serial dilution from 30 nM to 0.23 nM. (**B**) OV-90 cancer cell line was treated with PB-223 and the anti-HEL Human IgG1 isotype control. Both mAbs were added in a two-fold serial dilution from 60 nM to 0.47 nM. Data was recorded for 48 h and then automatically analyzed by data processing software installed on Incucyte (Gottingen, Germany). Each data point represents the mean of total integrated intensity (RCU × μm^2^/Image) value of two wells ± SEM of one experiment.

As shown in Figure 4A, Herceptin is internalized into SK-BR-3 cells in a dose-dependent manner after 48 h of incubation. When co-incubated with OV-90 cells, PB-223 showed internalization after 48 h in a dose-dependent manner. No internalization was observed with the anti-HEL Human IgG1 mAb, which was used as a negative control for the internalization assay (Figure 4B). NEO-102 was not compared with PB-223 in the assay.

## 4. Discussion

Since the development of hybridoma technology, mAbs have been used as therapy for the treatment of different types of diseases, including solid and liquid cancers, infectious diseases, immune diseases and inflammation [20].

Although mAbs showed promising efficacy in some cancers, there are still some limitations in their use as cancer immunotherapies, including the resistance of cancer cells (i.e., through the modulation of the expression of the antigen targeted by mAbs and immunosuppressive TME), the modest affinity of mAbs for their target antigens, and severe toxicities due to the fact that their target antigen is not expressed specifically by cancer cells but is also expressed by normal cells [20,21,22]. Current challenges to improving the efficacy and safety of mAbs include enhancing their affinity for their target antigen, developing mAbs with high specificity for targets expressed by cancer cells only, and engineering mAbs able to be internalized specifically into cancer cells to build more potent cytotoxic cancer drugs, such as ADCs [4,9,22,23].

In this study we have addressed most of these challenges. One possible reason for modest NEO-102 antitumor activity in a phase II study, performed on subjects with advanced refractory colorectal cancers, may be the low affinity of NEO-102 for its target antigen (KD of NEO-102 is 5.60 × 10^−9^ M) [15]. For this reason, we enhanced the affinity of NEO-102 for its target antigen, which is expressed by human cancers but not by healthy tissues [11]. This enhancement was achieved through affinity maturation using FASEBA technology [16]. We engineered the variable domains of heavy-chain (VH) and light-chain (VL) sequences of NEO-102 through FASEBA, with the aim of creating new clones with higher affinity (lower KD compared to NEO-102) for the NEO-102 target antigen.

Out of 2624 clones tested, the clone with the lowest KD and the highest increase in the KD ratio compared to NEO-102 was AHF18095 (PB-223). PB-223 exhibits a 4.55-fold improvement in binding affinity to BSM compared to NEO-102. One limitation of these results is that antibody concentrations in clone supernatants were not quantified and normalized prior to ELISA-based comparison during the affinity maturation process. In this case, differences in ELISA signal may partially reflect differences in antibody expression levels rather than affinity alone.

Next, we evaluated which glycans are specifically recognized by PB-223 and demonstrated that PB-223 binds to O-glycans terminating with α(2,6) sialic acids, with the strongest binding towards core 2 O-glycans containing a Neu5Ac(α2-6) Gal (β1-4) GlcNAc epitope in the β1-3 linkage. O-glycans are usually disrupted during carcinogenesis, and the presence of incomplete/truncated O-glycans attached to the proteins of cancer cells is associated with tumor progression, metastasis and poor prognosis [18]. Data obtained from this study showed that PB-223 has a stronger binding than NEO-102 towards two human cancer cell lines expressing core 2 O-glycans (a more than 2-fold increase in the percentage of positive cells in flow cytometry) and that it does not bind to human cancer cell lines not expressing core 2 O-glycans (e.g., the LOVO cell line). Although data from this study demonstrated the improved binding affinity of PB-223 to BSM compared to NEO-102, the affinity of PB-223 to the O-glycans attached to cancer proteins was not directly measured. Direct affinity determination using O-glycans or purified cancer-derived glycoprotein expressing O-glycans recognized by PB-223 would provide a more rigorous assessment of target-specific binding of PB-223 on cancer cell lines and would represent an important direction for future studies. In addition, another limitation is that we did not perform a direct glycan array comparison between PB-223 and the parental antibody NEO-102 in this study. The findings from this study do not definitively establish whether affinity maturation preserved or altered the complete epitope specificity of NEO-102. Future studies, including side-by-side profiling of O-glycans recognized by PB-223 and NEO-102, are needed to further define the effects of affinity maturation on epitope specificity.

In this study, we also demonstrated, via IHC, that PB-223 not only maintained the same strong binding toward several human malignant tumor tissues, including pancreas adenocarcinoma, rectum adenocarcinoma, lung adenocarcinoma, endometrioid adenocarcinoma, cervix squamous cell carcinoma, and high-grade serous carcinoma of the ovary, but that PB-223 also demonstrates an increase in the number of cancer tissues recognized by and/or in the IHC score compared to NEO-102 in prostate adenocarcinoma, colon adenocarcinoma, lung squamous cell carcinoma, HER2+ breast cancer and triple-negative breast cancer.

The observed increase was not statistically significant due to the low number of reactive tissues evaluated and to the heterogenicity of the staining toward tissues of the same histological origin. Further studies with bigger and more diverse panels of cancer tissues will be performed to determine whether the increase in the number of cancer tissues recognized and the higher IHC score observed with PB-223 compared to NEO-102 reach statistical significance.

In addition, we also observed that PB-223 can bind to other tumor tissues not recognized by NEO-102, such as pancreas neuroendocrine carcinoma.

Furthermore, we also demonstrated that PB-223 did not lose the specificity for cancer tissues after affinity maturation. Data obtained from the IHC analysis showed that PB-223 does not react to normal human brain, liver, lungs, colon, or lymph node tissues or to most normal human tissues adjacent to malignant tumors tested, including normal human tissues adjacent to malignant prostate, pancreas, lung, breast, uterus, ovary, kidney, and liver tumors. We observed that PB-223 reacted with goblet cells in normal human tissues adjacent to malignant colon and rectum tumors and with endocervical glandular cells in 1/3 of normal human tissues adjacent to a malignant cervical tumor. One of the reasons why PB-223 does not react with normal colon tissue but, instead, with goblet cells in the normal tissue adjacent to colon and rectum adenocarcinoma may be due to the fact that goblet cells increase the expression of heavily glycosylated mucins, such as MUC5AC, during carcinogenesis, leading to an overexpression of MUC5AC in a specific subtype of colorectal cancer called mucinous colorectal adenocarcinoma [24,25]. In addition, it has recently been demonstrated that expression of core 2 O-glycans in cancer cells, and not in normal cells, can differentiate colorectal cancer from the healthy colon epithelium [26].

It is possible that goblet cells in the normal tissue adjacent to colon and rectum adenocarcinomas transition into malignant cells with increased expression of core 2 O-glycans attached to MUC5AC. A similar phenomenon can explain the positivity of endocervical glandular cells in 1/3 of normal human tissues adjacent to a malignant cervical tumor. Overexpression of heavily glycosylated MUC5AC is usually found in most cases of endocervical adenocarcinomas [27,28]. Even in this case, it is possible that endocervical glandular cells in the normal tissue adjacent to a malignant cervical tumor transition into malignant cells with increased expression of core 2 O-glycans attached to MUC5AC. This is not the only possible explanation. For example, it is possible that there is a physiological expression of the PB-223 targeted glycoepitope in healthy mucin-producing epithelial cells, like goblet cells and endocervical glandular cells. Given the known complexity and heterogeneity of glycosylation patterns in secretory epithelial tissues, limited reactivity in certain normal cell populations cannot be excluded and warrants further investigation. Additional studies, using a larger panel of non-neoplastic healthy colon and cervix specimens, would be valuable to more comprehensively define the tissue distribution and specificity of the PB-223 target epitope.

One limitation of this study is that although the IHC protocol used to stain tissues from TMAs incorporated measures to minimize nonspecific backgrounds, a comparison between PB-223 or NEO-102 and an isotype-matched control antibody was not performed. Future studies, incorporating isotype controls in TMAs, will provide additional confirmation of PB-223 specificity in IHC.

Additionally, this study demonstrated that PB-223 can be internalized in the human ovarian cancer cell line OV-90, which expresses its target O-glycans. The absence of internalization with the isotype control antibody confirms that uptake is target-driven rather than nonspecific. This suggests a potential application of PB-223 for targeted drug delivery. However, internalization was not evaluated in cancer cell lines with no or low reactivity with PB-223 in flow cytometry. Future studies comparing PB-223 positive and PB-223 negative cell lines in flow cytometry will be important to further establish that PB-223 internalization is driven by antigen expression rather than nonspecific cellular uptake.

It is important to mention that while the NEO-102 target antigen was originally identified as a cancer-specific glycosylated variant of MUC5AC, selectively expressed in colorectal and pancreatic cancers and with minimal expression in normal tissues, the glycan array results provided in this study suggest that PB-223 recognizes a tumor-associated glycan motif rather than the MUC5AC protein backbone itself. Glycan array, flow cytometry and IHC data reported in this study suggest that O-glycans recognized by PB-223 are not exclusively attached to MUC5AC, but they can be shared across multiple glycoproteins expressed specifically on cancer cells. Additional studies are required to fully define all cancer-associated proteins that carry O-glycans recognized by PB-223. Furthermore, additional studies, such as glycosidase treatment, glycosyltransferase perturbation, and glycoengineered cellular models, are required to define the precise glycan epitope recognized by PB-223.

Moreover, the specific molecular mechanisms by which the selected mutations of VH and VL regions enhance glycan recognition and reduce dissociation kinetics remain to be determined. Future structural studies, including sequence-structure analyses and molecular modeling, need to be performed to define the contribution of individual mutations to affinity maturation.

The evidence—that PB-223 has the ability to bind specifically to a wide variety of human carcinomas but does not bind to healthy human tissues or most normal human tissues adjacent to malignant tumors and that it can be internalized into a human cancer cell line expressing its target antigen—provides a potential rationale for using PB-223 as an mAb to build an ADC.

A critical aspect of ADC development is identifying a target antigen expressed specifically by cancer cells with minimal or no expression in healthy tissues. It should also be present on the surface of tumor cells for optimal accessibility and be capable of internalizing upon mAb binding to enable intracellular delivery of the payload [20,23]. Cancer-specific O-glycans recognized by PB-223 can be a valid target for improving tumor selectivity and reducing toxicity relative to protein antigens that may be expressed in normal tissues [18,29]. Furthermore, efficient internalization of ADC following antibody–antigen engagement is a critical requirement for the efficacy of ADCs and other payload-based antibody therapies [23,30,31]. The observed internalization kinetics of PB-223 also provide a rationale for selecting linker–payload combinations optimized for lysosomal processing, which will be important for maximizing therapeutic efficacy while minimizing systemic toxicity [23,31].

Although both PB-223 and NEO-102 exhibit nanomolar affinity, a 4.55-fold increase in affinity may still provide functional advantages, including enhanced tumor cell binding, increased retention on target cells, and improved internalization, all of which may be beneficial for antibody-based therapeutic applications such as ADC.

We are currently testing the in vitro and in vivo efficacy of a PB-223 based ADC against human carcinomas expressing O-glycans recognized by PB-223. Preliminary data were presented at the Society for Immunotherapy of Cancer (SITC) Annual Meeting in 2025 and showed that the PB-223 based ADC, PB-vc-MMAE-5, induced a promising tumor growth inhibition, compared to control groups, in vivo, in NOD-SCID mice bearing OV-90 xenografts, with minimal toxicity [32].

Although the data presented in this study and the preliminary data on the preclinical activity of PB-223 based ADC are encouraging, there are potential limitations to using PB-223 to build an effective ADC. For example, the high affinity of PB-223 for its target antigen may reduce tumor penetration through a binding-site barrier effect and may also increase binding to low-level antigen expression in normal tissues, thereby narrowing the therapeutic window. This issue is especially relevant for glycan-targeting antibodies because the recognized glycan structures may not be completely tumor-restricted and may potentially be shared among multiple glycoproteins. Further studies evaluating tumor penetration, biodistribution, therapeutic index, and safety of PB-223 based ADCs will be required to determine whether the observed affinity improvement translates into an optimal therapeutic benefit.

In addition, although the pH-sensitive internalization assay demonstrates uptake of PB-223 into acidic intracellular compartments, which is consistent with lysosomal trafficking, it does not provide clear evidence of whether the antibody undergoes productive lysosomal trafficking, degradation, or recycling back to the cell surface. Since effective ADC activity depends strongly on lysosomal delivery and payload release, additional studies, including comparative internalization kinetics between NEO-102 and PB-223, co-localization with lysosomal markers, receptor recycling analyses, degradation studies, and ADC cytotoxicity assessments, need to be performed to further evaluate the benefit of PB-223 as an ADC carrier.

Beyond ADC development, PB-223 could also be explored in alternative therapeutic formats, including bispecific antibodies and immune-engaging constructs [22,33].

In addition, the enhanced binding affinity and robust internalization properties observed for PB-223 highlight its translational promise as a candidate for radioimmunotherapy (RIT). Although radioligand therapies with mAbs can be effective even without mAb internalization into target cells, their internalization through mAbs can enhance their efficacy, since internalized antibodies can retain radionuclides intracellularly, thereby increasing radiation dose deposition within tumor cells and improving therapeutic efficacy [34,35,36].

Importantly, several studies have demonstrated that antibodies targeting tumor-associated carbohydrate or glycan epitopes can be successfully engineered for radionuclide conjugation, achieving favorable tumor uptake and prolonged retention [37,38,39].

However, the use of PB-223 to build a bispecific antibody, radiopharmaceutical, or other therapeutic formats is not directly supported by the data presented in this study and remains hypothetical. Future studies, including conjugate generation, cytotoxicity testing, pharmacokinetic evaluation, and in vivo efficacy and safety investigations, need to be performed to assess the potential of PB-223 to be further developed in other immunotherapeutic formats.

## 5. Conclusions

The findings of this study support PB-223 as a promising tumor-selective mAb targeting a cancer-specific glycosylation epitope, with potential applicability across multiple solid tumor indications with the potential for optimal efficacy and minimal toxicity. These findings also highlight the potential use of glycan expression as a predictive biomarker for patient selection. Knowing the correct marker to choose the most effective treatment is becoming a crucial strategy in the era of precision oncology [40]. The heterogeneity of PB-223’s staining patterns, observed across tumor types and individual samples, is consistent with known variability in tumor glycosylation profiles [41,42].

While glycan-targeting antibodies offer potential advantages, including recognition of cancer-associated glycosylation patterns that may be shared across multiple tumor types, several challenges remain, including antigen heterogeneity, variable tumor accessibility, and the possibility of low-level expression in normal tissues. Therefore, although the properties of PB-223 support its further evaluation as a therapeutic candidate, the therapeutic applicability of this antibody requires additional preclinical and clinical validation. The results presented here should therefore be viewed as providing a foundation for future therapeutic development rather than definitive evidence of clinical efficacy.

In this regard, the data presented in this study underscores the importance of biomarker-driven clinical development strategies and suggests that patient stratification based on glycan expression may be critical for maximizing PB-223 clinical benefit.

Future translational efforts should focus on validating the expression of O-glycans recognized by PB-223 in large patient cohorts, correlating glycan expression with clinical outcomes. These studies will be essential for informing patient selection strategies and dose optimization during early-phase clinical trials.

## Figures and Tables

**Table 1:** Residues selected for PML library construction.

CDRs	VH-CDR1	VH-CDR2	VH-CDR3	VL-CDR1	VL-CDR2	VL-CDR3
Residue No.	26-35	50-65	98-102	24-33	49-55	88-96
Labeled name	AA1-10	AA11-26	AA27-31	AA1-10	AA11-17	AA18-26

**Table 2:** Residues selected for PML library construction and considered “beneficial mutants” for improved binding affinity.

Region	Mutation
VL-54A	G
VH-28S	E,V
VH-35N	I
VH-58S	G,V
VH-61S	L
VH-62G	K

**Table 3:** Characteristics of mutant clones with best affinity for BSM in ELISA.

NO.	Sample ID	OD450 nm (Mutant)	OD450 nm (Parental)	Ratio = OD (Mutant)/ OD(Parental)	Sequence ID	VH					VL
						28	35	58	61	62	54
					WT	S	N	S	S	G	A
1	P01-B01	0.645	0.404	1.599	AHF18093	E	.	V	.	K	G
2	P01-H02	0.655	0.404	1.623	AHF18094	V	I	V	.	.	.
3	P01-B04	0.663	0.404	1.643	AHF18095	E	I	V	.	K	G
4	P01-F07	0.664	0.404	1.646	AHF18096	E	I	V	.	.	.
5	P01-A09	0.639	0.404	1.584	AHF18097	V	I	V	.	.	.
6	P02-H03	0.656	0.417	1.573	AHF18098	E	I	V	L	K	.
7	P02-H07	0.661	0.417	1.585	AHF18099	E	I	V	.	K	.
8	P02-H08	0.677	0.417	1.624	AHF18100	E	I	V	.	K	.
9	P02-A11	0.638	0.417	1.530	AHF18101	E	I	V	L	K	.
10	P02-H11	0.683	0.417	1.638	AHF18102	.	I	V	.	.	.
11	P03-F01	0.664	0.409	1.625	AHF18103	.	I	V	L	.	.
12	P03-A06	0.649	0.409	1.589	AHF18104	E	I	.	L	K	.
13	P03-B06	0.632	0.409	1.547	AHF18105	.	I	V	.	.	.
14	P03-C06	0.665	0.409	1.628	AHF18106	E	I	V	L	.	.
15	P03-A12	0.689	0.409	1.687	AHF18107	V	I	V	.	.	.

The table shows the OD ratio between the mutant clones and the parental clone in ELISA and specific aminoacidic mutants in the VH and VL regions from each clone. A = alanine, E = glutamic acid, G = glycine, K = lysine, I = isoleucine, L = leucine, N = asparagine, S = serine, V = valine. Dots mean no mutation compared to WT.

**Table 4:** Binding kinetics to BSM of 15 clones sent for affinity rankings compared to NEO-102.

NO.	Clone	ka (1/Ms)	kd (1/s)	KD (M)	Increase Ratio (KD)
1	AHF18093	1.56 × 10^5^	2.19 × 10^−4^	1.40 × 10^−9^	4.00
2	AHF18094	1.55 × 10^5^	5.01 × 10^−4^	3.24 × 10^−9^	1.73
**3**	**AHF18095** (**PB-223**)	**1.59** × **10^5^**	**1.95** × **10^−4^**	**1.23** × **10^−9^**	**4.55**
4	AHF18096	1.57 × 10^5^	2.27 × 10^−4^	1.44 × 10^−9^	3.89
5	AHF18097	1.51 × 10^5^	3.54 × 10^−4^	2.34 × 10^−9^	2.39
6	AHF18098	1.13 × 10^5^	4.36 × 10^−4^	3.87 × 10^−9^	1.45
7	AHF18099	1.32 × 10^5^	2.64 × 10^−4^	2.00 × 10^−9^	2.80
**8**	**AHF18100**	**1.48** × **10^5^**	**2.02** × **10^−4^**	**1.37** × **10^−9^**	**4.09**
9	AHF18101	1.25 × 10^5^	3.10 × 10^−4^	2.47 × 10^−9^	2.27
10	AHF18102	1.38 × 10^5^	2.66 × 10^−4^	1.93 × 10^−9^	2.90
11	AHF18103	1.25 × 10^5^	3.95 × 10^−4^	3.15 × 10^−9^	1.78
**12**	**AHF18104**	**1.32** × **10^5^**	**1.93** × **10^−4^**	**1.46** × **10^−9^**	**3.84**
13	AHF18105	1.29 × 10^5^	2.85 × 10^−4^	2.21 × 10^−9^	2.53
14	AHF18106	1.28 × 10^5^	3.23 × 10^−4^	2.53 × 10^−9^	2.21
15	AHF18107	1.16 × 10^5^	3.21 × 10^−4^	2.78 × 10^−9^	2.01
	U6901-WT1 (NEO-102)	9.63 × 10^4^	5.40 × 10^−4^	5.60 × 10^−9^	

Antibodies were captured on the sensor chip. The target antigen BSM was used as the analyte. The data for the dissociation (kd) and association (ka) rate constants were obtained using Biacore evaluation software (version 3.2). The equilibrium dissociation constants (KD) were calculated from the ratio of kd over ka. Clones depicted in bold are clones with a higher increase in the KD ratio compared to the wild-type clone (NEO-102). These clones were selected for antibody production and purification. The increase ratio was calculated in the following manner: KD of U6901-WT1 (NE0-102)/KD of mutant clones. The clone AHF18095 was then selected for further experiments and was designated PB-223. U6901-WT1 is the wild-type mAb NEO-102.

**Table 5:** Binding profile of PB-223 vs. NEO-102 in different human cancer cell lines according to flow cytometry.

Cell Line	Tumor Type	% NEO-102 Positive Cells ± SD (MFI) *	% PB-223 Positive Cells ± SD (MFI) *	*p* Value	Fold Increase in % of Positive Cells (PB-223 vs. NEO-102)	Most Abundant O-Glycan Recognized by PB-223 (% Expression)
LoVo	Colorectal adenocarcinoma; Dukes’ type C, grade IV	0.37 ± 0.13 (0)	0.60 ± 0.30 (0)	NS	1.62	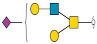 O53 (0.29%)
SW-480	Colorectal adenocarcinoma; Dukes’ type B	0.29 ± 0.08 (0)	0.29 ± 0.03 (0)	NS	1.00	N.T.
**SW-403**	Colorectal adenocarcinoma; Dukes’ type C, grade III	**26.09** ± 11.85 (31)	**57.97** ± 15.89 (93)	**NS**	**2.22**	N.T.
**COLO 205**	Colorectal adenocarcinoma; Dukes’ type D	6.3 ± 4.93 (13)	**24.94** ± 22.05 (31)	**NS**	**3.96**	N.T.
**HCC1937**	Ductal Triple negative breast cancer; Stage IIB, grade 3	**19.38** ± 18.58 (40)	**52.04** ± 17.13 (101)	**NS**	**2.69**	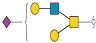 O53 (6.32%)
**OV-90**	Malignant Papillary Serous Adenocarcinoma of ovary	**25.13** ± 1.27 (142)	**43.38** ± 0.98 (215)	**0.0038**	**2.15**	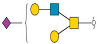 O53 (6.76%)
DU 145	Prostate adenocarcinoma; grade II	2.63 ± 0.96 (14)	2.28 ± 0.76 (13)	NS	0.87	N.T.
PC-3	Prostate adenocarcinoma; Grade IV	5.50 ± 3.43 (30)	8.38 ± 5.61 (39)	NS	1.52	N.T.

The table depicts the percentage of viable cells ± SD reactive with PB-223 compared to NEO-102 and, in brackets, the MFI (median fluorescence intensity) of PB-223 or NEO-102 positive cells. * For each cell line, results are presented as mean ± SD of two independent experiments. In each experiment, the percentage of PB-223 or NEO-102 positive cells was calculated with the following formula: % PB-223 or NEO-102 stained cells − % unstained cells. In each experiment, the MFI of PB-223 or NEO-102 positive cells was calculated with the following formula: MFI of PB-223 or NEO-102 stained cells − MFI of unstained cells. Difference in percentage of positive cells reactive with PB-223 compared to NEO-102 was considered statistically significant with a *p* value < 0.05, using an unpaired T-test. Cells with staining values > 10% were considered positive. PB-223 and NEO-102positive cell lines appear in bold text. N.T.: not tested; NS: not significant; SD: standard deviation.

**Table 6:** Comparison of reactivity of PB-223 vs. NEO-102 in different human malignant tumor tissues according to IHC.

Human Cancer	mAb	Tissues Positive	IHC Score 1 (% Positive Cells)	IHC Score 2 (% Positive Cells)	IHC Score 3 (% Positive Cells)
Prostate (adenocarcinoma)	PB-223	3/11 (27%)	1 (30%)	1 (60%)	1 (90%)
	NEO-102	2/11 (18%)	1 (20%)		1 (90%)
Pancreas (adenocarcinoma)	PB-223	9/10 (90%)	1 (50%)		8 (94%) *
	NEO-102	9/10 (90%)	1 (30%)		8 (94%) *
Pancreas (neuroendocrine carcinoma)	PB-223	1/1(100%)	1 (80%)		
	NEO-102	0/1 (0%)			
Colon (adenocarcinoma)	PB-223	10/11 (91%)	3 (27%) *	1 (10%)	6 (72%) *
	NEO-102	7/11 (64%)		1 (10%)	6 (68%) *
Rectum (adenocarcinoma)	PB-223	3/3 (100%)		1 (20%)	2 (100%) *
	NEO-102	3/3 (100%)	1 (10%)		2 (100%) *
Lung (squamous cell carcinoma)	PB-223	2/7 (29%)		1 (60%)	1 (50%)
	NEO-102	2/7 (29%)	1 (30%)		1 (30%)
Lung (adenocarcinoma)	PB-223	3/4 (75%)		1 (15%)	2 (100%) *
	NEO-102	3/4 (75%)		1 (5%)	2 (80%) *
Breast (invasive carcinoma HER2+)	PB-223	14/54 (26%)	3 (83%) *	7 (83%) *	4 (80%) *
	NEO-102	14/54 (26%)	6 (80%) *	3 (70%) *	5 (82%) *
Breast (invasive triple negative)	PB-223	2/28 (7%)		1 (15%)	1 (100%)
	NEO-102	2/28 (7%)			2 (53%) *
Uterus (endometrioid adenocarcinoma)	PB-223	4/8 (50%)	1 (10%)	1 (30%)	2 (75%) *
	NEO-102	4/8 (50%)	1 (5%)	2 (30%) *	1 (80%)
Cervix (squamous cell carcinoma)	PB-223	1/3 (33%)		1 (25%)	
	NEO-102	1/3 (33%)		1 (20%)	
Ovary (high-grade serous carcinoma)	PB-223	2/3 (67%)		2 (23%) *	
	NEO-102	2/3 (67%)		2 (15%) *	
Kidney (clear cell carcinoma)	PB-223	0/11 (0%)			
	NEO-102	0/11 (0%)			
Esophagus (squamous cell carcinoma)	PB-223	0/3 (0%)			
	NEO-102	0/3 (0%)			
Stomach (adenocarcinoma)	PB-223	0/3 (0%)			
	NEO-102	0/3 (0%)			
Liver (hepatocellular carcinoma)	PB-223	0/3 (0%)			
	NEO-102	0/3 (0%)			

The table depicts the IHC profile of PB-223 compared to NEO-102 staining of human malignant tumor microarray tissues. For each tissue, the percentage of cells reactive with PB-223 compared to NEO-102 and the IHC score of PB-223 or NEO-102 positive cells are reported. IHC score was determined using a semi-quantitative scale: score 1, weak staining; score 2, moderate staining; and score 3, strong staining. Tissues with an IHC score of 0 were considered negative for the expression of the antigen recognized by NEO-102 and PB-223. Scores were assigned based on the intensity and extent of membranous/cytoplasmic staining observed in the tissue sections, and the extent of positive cells within each tissue section IHC score was determined by a review of stained tissues from a single board-certified pathologist blinded to antibody identity. * Results are presented as the mean of positive cells between several tissue microarrays with the same histology.

## Data Availability

All data generated or analyzed during this study are included in this published article and its Supplementary Information Files. Data are available from the corresponding author upon reasonable request.

## Supplementary Materials

The following supporting information can be downloaded at https://www.mdpi.com/article/10.3390/cancers18142336/s1. Figure S1: Comparison of binding curves of NEO-102 and PB-223 in flow cytometry for each cancer cell line tested in each experiment. Each experiment provides the curve of unstained cells compared to curves of cells stained with PB-223, NEO-102 and secondary antibodies alone. New NEO-102 is PB-223; REF STD NEO-102 is NEO-102; IgG1 AF488 is the mouse anti-human IgG1 Fc secondary antibody conjugated with the AF488 fluorescent dye. Figure S2: Monoisotopic mass, proposed compositions, proposed structures, and relative abundance of O-glycan expressed by each cell line tested. File S1: supplementary materials and methods.

## Abbreviations

The following abbreviations are used in this manuscript:

ADC	Antibody–drug conjugate
ADCC	Antibody-dependent cellular cytotoxicity
ATCC	American Type Culture Collection
BSM	Bovine Submaxillary Mucin
ELISA	Enzyme-linked immunosorbent assay
FASEBA	Fast Screening for Expression, Biophysical Properties, and Affinity
FFPE	Formalin-fixed paraffin-embedded
GABB	Glycan Array Blocking Buffer
HC	Heavy chain
IHC	Immunohistochemistry
IPTG	Isopropil-β-D-1-tiogalattopiranoside
KA	Association constant
KD	Dissociation constant
LC	Light chain
mAb	Monoclonal antibody
NGS	Next-generation sequencing
PBS	Phosphate-buffered saline
PCR	Polymerase Chain Reaction
PML	Precise mutagenesis library
PSMA	Prostate-specific membrane antigen
RFU	Relative fluorescence unit
RIT	Radioimmunotherapy
RT	Room temperature
SITC	Society for Immunotherapy of Cancer
SOP	Standard operating procedure
TAAs	Tumor-associated antigens
TBS	Tris-buffer saline
TBST	Tris-buffer saline with Tween 20
TMA	Tissue microarray
TME	Tumor microenvironment
VH	Variable heavy-chain
VL	Variable light-chain
