# Supplementary material for: Affinity Maturation and Characterization of the Novel Monoclonal Antibody (mAb) PB-223 Targeting Cancer-Specific O-Glycans Terminating with α(2,6) Sialic Acids
Source: Cancers (Basel). 2026 Jul 20;18(14):2336. doi: 10.3390/cancers18142336 (PMC13406998; doi:10.3390/cancers18142336)
Supplement: Supplementary file 1 [file cancers-18-02336-s001.zip › File S1-supplementary materials and methods/human-on-human HRP polymer kit section 2.6.pdf]

Human-on-Human HRP-Polymer

Human Antibodies on Human Tissues
902-4056K-052620

BIOCARE MEDICAL

**Catalog Number:**	**BRR 4056K G, H**
**Description:**	6.0, 25 mL

Intended Use:
For Research Use Only. Not for use in diagnostic procedures.

Summary & Explanation:
Humanized antibodies are an improving and rapidly growing class of therapeutics for treatment of human disease. Their efficacy depends highly on specificity and sensitivity for the desired target antigen. Thus, preclinical studies of potential human (and humanized) antibody therapeutics include immunohistochemical (IHC) screening. However, non-specific staining due to endogenous human IgG binding is a technical problem in human antibody detection. The Human-on-Human HRP-Polymer kit delivers a solution to this problem by providing reagents to tag the human primary antibody of interest with digoxigenin, even in the presence of other non-IgG proteins, and detect it on human tissue with an HRP-polymer system. With this detection system, human antibodies bound to human tissue can be detected with high specificity and sensitivity, while significantly reducing or eliminating background. Additionally, the Human-on-Human HRP-Polymer kit does not require overnight incubation steps, allowing for rapid screening of multiple human primary antibody clones.

Known Applications:
Formalin-fixed paraffin-embedded (FFPE) tissues

Supplied As:

6 mL Kit
Digoxigenin anti-Human Linker (BRR4053B) 0.5 mL
Human Absorption Reagent (BRR4054B) 0.5 mL
Mouse anti-Digoxigenin Secondary (BRR4055G) 6 mL
MACH 2 Mouse HRP-Polymer (MHRP520G) 6 mL

25 mL Kit
Digoxigenin anti-Human Linker (BRR4053D) 2.1 mL
Human Absorption Reagent (BRR4054D) 2.1 mL
Mouse anti-Digoxigenin Secondary (BRR4055H) 25 mL
MACH 2 Mouse HRP-Polymer (MHRP520H) 25 mL

Materials and Reagents Needed But Not Provided:
Microscope slides, positively charged
Desert Chamber* (Drying oven)
Positive and negative tissue controls
Xylene (Could be substituted with xylene substitute*)
Ethanol or reagent alcohol
Decloaking Chamber* (Pressure cooker)
Deionized or distilled water
Wash buffer*
Pretreatment reagents*
Enzyme digestion*
Peroxidase block*
Protein block*
Primary antibody*
Negative control reagents*
Chromogens*
Hematoxylin*
Bluing reagent*
Mounting medium*

* Refer to the Biocare Medical website located at http://biocare.net for information regarding catalog numbers and ordering.

Species Reactivity:
Human IgG heavy and light chains

Storage and Stability:
Store kit at 2ºC to 8ºC. The product is stable to the expiration date printed on the label, when stored under these conditions. Do not use after expiration date.

Staining Protocol Recommendations:

Preparation of the Digoxigenin-Tagged Antibody (1 mL) (Figure 1):
1. Combine 80 µL of Digoxigenin anti-Human Linker with primary antibody (0.1-0.5 µg in 200-400 µL of diluent) and vortex for 5 seconds.
2. Incubate mixture at room temperature (RT) for 1 hour.
3. Add 80 µL of Human Absorption Reagent to mixture, vortex for 5 seconds, and incubate for 30 minutes at RT.
4. Add desired antibody diluent to a final volume of 1 mL.

The primary antibody is now tagged with digoxigenin and is ready to use. Shelf life may vary and must be determined by the individual investigator.
Note: Instructions are for 1 mL of tagged antibody. Preparation can be scaled linearly (i.e. for 0.5 mL of tagged antibody, divide all quantities by 2).

Staining Procedure:
1. Deparaffinize tissue sections in Slide Brite or xylene. Hydrate slides in a graded series of alcohol to water.
2. Apply Peroxidazed 1 for 5 minutes at RT.
3. Rinse slides in deionized (DI) water.
4. Pretreatment solution/protocol:
Heat-retrieval (optional): Heat slides in Biocare's Diva Decloaker, Reveal Decloaker, or Borg Decloaker using Biocare's Decloaking Chamber. Proteolytic digestion (optional): Digest tissue with Trypsin or Pepsin.
5. Rinse slides with two changes of TBS wash buffer.
6. Protein block (optional): Apply Biocare's Background Punisher for 10 minutes at RT.
7. Rinse slides with TBS wash buffer.
8. Apply digoxigenin-tagged primary antibody to tissue and incubate for 30 minutes to 1 hour at RT.
9. Rinse slides with TBS wash buffer.
10. Apply Mouse anti-Digoxigenin Secondary to tissue and incubate for 15 minutes at RT.
11. Rinse slides with TBS wash buffer.
12. Apply MACH 2 Mouse HRP-Polymer to tissue and incubate at RT for 30 minutes.
13. Rinse slides with TBS wash buffer for 5 minutes.
14. Apply DAB for 5 minutes at RT. Wash in DI water.
15. Apply CAT Hematoxylin for 30 seconds to 1 minute or Tacha's Automated Hematoxylin for 5 minutes. Wash in DI water.
16. Apply Tacha's Bluing Solution for 1-2 minutes. Wash in DI water.
17. Dehydrate, clear and coverslip.

Staining Protocol Recommendations (VALENT® Automated Slide Staining Platform):
Human-on-Human HRP-Polymer is compatible for use with the VALENT. Refer to the User Manual for specific instructions for use. Protocol parameters in the Protocol Manager should be programmed as follows:
1. **Deparaffinization:** Deparaffinize for 8 minutes with Val DePar.
2. **Pretreatment:** Perform heat retrieval at 98°C for 60 minutes using Val AR-Lo pH, 5X (use at 1X).
3. **Peroxidase Block:** Block for 5 minutes with Val Peroxidase Block.
4. **Protein Block:** Incubate for 10 minutes at RT with Val Background Block.
5. **Primary Antibody:** Incubate for 45 minutes with digoxigenin-tagged primary antibody.
6. **Secondary:** Incubate for 30 minutes with Mouse anti-Digoxigenin Secondary.
7. **Polymer:** Incubate for 45 minutes with MACH2 Mouse HRP-Polymer.

Biocare Medical
60 Berry Drive
Pacheco, CA 94553
USA

Staining Protocol Recommendations (VALENT® Automated Slide Staining Platform) Cont'd:

8. **Chromogen:** Incubate for 5 minutes with Val DAB.
9. **Counterstain:** Counterstain for 5 minutes with Val Hematoxylin.

Technical Note:
This kit has been tested with Biocare's Renaissance Background Reducing Diluent. The optimal diluent is dependent on the primary antibody and must be determined by the investigator.

Limitations:
This product is provided for Research Use Only (RUO) and is not for use in diagnostic procedures. Suitability for specific applications may vary and it is the responsibility of the end user to determine the appropriate application for its use.

Precautions:
1. Materials included in this kit contain non-hazardous human biological material. Please use the appropriate personal protection equipment when handling materials.
2. This product is not classified as hazardous. The preservative used in kit reagents is Proclin 950. The concentration of Proclin (less than 0.25%) does not meet the OSHA criteria for hazardous substances. Overexposure to Proclin can cause skin and eye irritation and irritation to mucous membranes and upper respiratory tract. Wear disposable gloves when handling reagents.
3. Specimens, before and after fixation, and all materials exposed to them should be handled as if capable of transmitting infection and disposed of with proper precautions. Never pipette reagents by mouth and avoid contacting the skin and mucous membranes with reagents and specimens. If reagents or specimens come in contact with sensitive areas, wash with copious amounts of water.
4. Microbial contamination of reagents may result in an increase in nonspecific staining.
5. Incubation times or temperatures other than those specified may give erroneous results. The user must validate any such change.
6. Do not use reagent after the expiration date printed on the vial.
7. The SDS is available upon request and is located at http://biocare.net.
8. Consult OSHA, federal, state or local regulations for disposal of any toxic substances.
9. Proclin™ is a trademark of Rohm and Haas Company, or of its subsidiaries or affiliates.

Technical Support:
Contact Biocare's Technical Support at 1-800-542-2002 for questions regarding this product.

Troubleshooting Guide:

No Staining
1. Critical reagent (such as primary antibody) omitted.
2. Staining steps performed incorrectly or in the wrong order.
3. Heat-induced epitope retrieval (HIER) step was performed incorrectly using the wrong time, the wrong order or the wrong pretreatment.
4. Insufficient amount of antigen.
5. Primary antibody incubation period too short.
6. Improperly mixed substrate and/or chromogen solution(s).

Weak Staining
1. Tissue is either over-fixed or under-fixed.
2. Primary antibody incubation too short.
3. Low expression of antigen.
4. Heat-induced epitope retrieval (HIER) steps performed incorrectly using wrong time, in the wrong order, or the wrong pretreatment.
5. Over-development of substrate.
6. Excessive rinsing during wash steps.
7. Omission of critical reagent.
8. Incorrect procedure in reagent preparation.
9. Improper procedure in test steps.

Non-specific or High Background Staining
1. Tissue is either over-fixed or under-fixed.
2. Incorrect blocking reagent used; blocker should be from same species in which the secondary antibody was raised.
3. Tissue may need a longer or a more specific protein block.
4. Substrate is overly-developed.
5. Tissue was inadequately rinsed.
6. Deparaffinization incomplete.
7. Tissue damaged or necrotic.

Tissues Falling Off
1. Slides were not positively charged.
2. A slide adhesive was used in the waterbath.
3. Tissue was not dried properly.
4. Tissue contained too much fat.

Specific Staining Too Dark
1. Concentrated antibody not diluted out properly (being used at too high of a concentration).
2. Incubation of primary antibody or detection too long.

Figure 1:

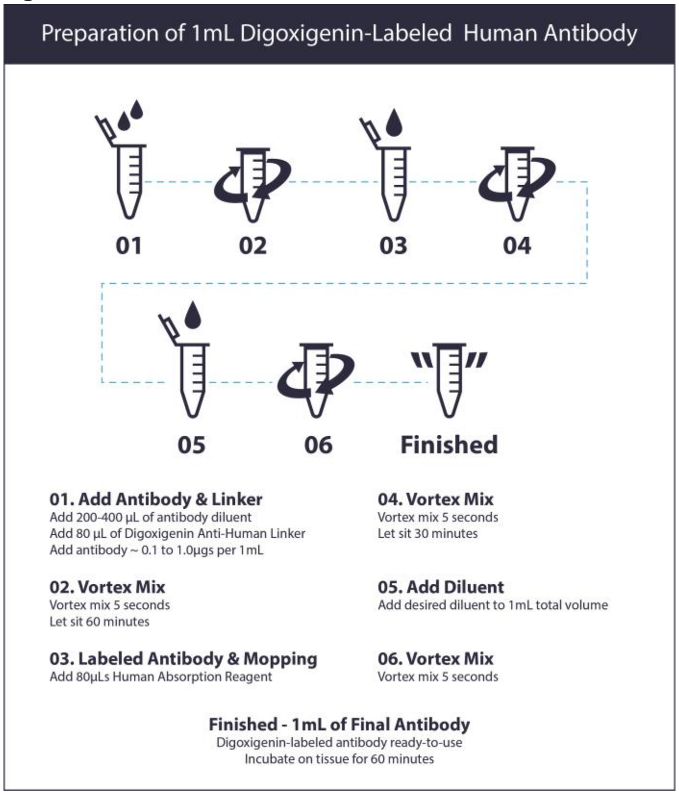

Biocare Medical
60 Berry Drive
Pacheco, CA 94553
USA

Tel: 800-799-9499 | www.biocare.net | Fax: 925-603-8080
