# Supplementary material for: Affinity Maturation and Characterization of the Novel Monoclonal Antibody (mAb) PB-223 Targeting Cancer-Specific O-Glycans Terminating with α(2,6) Sialic Acids
Source: Cancers (Basel). 2026 Jul 20;18(14):2336. doi: 10.3390/cancers18142336 (PMC13406998; doi:10.3390/cancers18142336)
Supplement: Supplementary file 1 [file cancers-18-02336-s001.zip › File S1-supplementary materials and methods/protocol details IHC section 2.6.pdf]

Assay Information

Antibody

Target ID	Target	Vendor	Cat #	Species	Clone
N/A	PB-223	BosterBio	PB-223	Human	AHF18095
N/A	NEO-102	BosterBio	NEO-102	Human	N/A

Assay Conditions

Platform	Target	Sample	Antibody	Epitope Retrieval	Primary Ab Incubation	Ab Dilution	Detection	Protocol ID*
Leica Bond Rx	PB-223	BCN601a-1	PB-223	DIVA Soln for 20m (110C)	1hr Ab Incubation (RT)	1 ug/mL	Biocare Human-on-Human HRP Detection System	Manual IHC (Biocare Hs-on-Hs-A01)
Leica Bond Rx	PB-223	BCN721b-1	PB-223	DIVA Soln for 20m (110C)	1hr Ab Incubation (RT)	1 ug/mL	Biocare Human-on-Human HRP Detection System	Manual IHC (Biocare Hs-on-Hs-A01)
Leica Bond Rx	PB-223	BR1102-1	PB-223	DIVA Soln for 20m (110C)	1hr Ab Incubation (RT)	1 ug/mL	Biocare Human-on-Human HRP Detection System	Manual IHC (Biocare Hs-on-Hs-A01)
Leica Bond Rx	PB-223	nHMT25-01A	PB-223	DIVA Soln for 20m (110C)	1hr Ab Incubation (RT)	1 ug/mL	Biocare Human-on-Human HRP Detection System	Manual IHC (Biocare Hs-on-Hs-A01)
Leica Bond Rx	PB-223	OV90 & cHMT25-01A	PB-223	DIVA Soln for 20m (110C)	1hr Ab Incubation (RT)	1 ug/mL	Biocare Human-on-Human HRP Detection System	Manual IHC (Biocare Hs-on-Hs-A01)

See next page for protocol details

Assay Information

Assay Conditions

Platform	Target	Sample	Antibody	Epitope Retrieval	Primary Ab Incubation	Ab Dilution	Detection	Protocol ID*
Leica Bond Rx	NEO-102	BCN601a-1	NEO-102	DIVA Soln for 20m (110C)	1hr Ab Incubation (RT)	1 ug/mL	Biocare Human-on-Human HRP Detection System	Manual IHC (Biocare Hs-on-Hs-A01)
Leica Bond Rx	NEO-102	BCN721b-1	NEO-102	DIVA Soln for 20m (110C)	1hr Ab Incubation (RT)	1 ug/mL	Biocare Human-on-Human HRP Detection System	Manual IHC (Biocare Hs-on-Hs-A01)
Leica Bond Rx	NEO-102	BR1102-1	NEO-102	DIVA Soln for 20m (110C)	1hr Ab Incubation (RT)	1 ug/mL	Biocare Human-on-Human HRP Detection System	Manual IHC (Biocare Hs-on-Hs-A01)
Leica Bond Rx	NEO-102	nHMT25-01A	NEO-102	DIVA Soln for 20m (110C)	1hr Ab Incubation (RT)	1 ug/mL	Biocare Human-on-Human HRP Detection System	Manual IHC (Biocare Hs-on-Hs-A01)
Leica Bond Rx	NEO-102	OV90 & cHMT25-01A	NEO-102	DIVA Soln for 20m (110C)	1hr Ab Incubation (RT)	1 ug/mL	Biocare Human-on-Human HRP Detection System	Manual IHC (Biocare Hs-on-Hs-A01)
Leica Bond Rx	Negative Control	BCN601a-1	Secondary Only	DIVA Soln for 20m (110C)	1hr Ab Incubation (RT)	1 ug/mL	Biocare Human-on-Human HRP Detection System	Manual IHC (Biocare Hs-on-Hs-A01)
Leica Bond Rx	Negative Control	BCN721b-1	Secondary Only	DIVA Soln for 20m (110C)	1hr Ab Incubation (RT)	1 ug/mL	Biocare Human-on-Human HRP Detection System	Manual IHC (Biocare Hs-on-Hs-A01)
Leica Bond Rx	Negative Control	BR1102-1	Secondary Only	DIVA Soln for 20m (110C)	1hr Ab Incubation (RT)	1 ug/mL	Biocare Human-on-Human HRP Detection System	Manual IHC (Biocare Hs-on-Hs-A01)
Leica Bond Rx	Negative Control	nHMT25-01A	Secondary Only	DIVA Soln for 20m (110C)	1hr Ab Incubation (RT)	1 ug/mL	Biocare Human-on-Human HRP Detection System	Manual IHC (Biocare Hs-on-Hs-A01)
Leica Bond Rx	Negative Control	OV90 & cHMT25-01A	Secondary Only	DIVA Soln for 20m (110C)	1hr Ab Incubation (RT)	1 ug/mL	Biocare Human-on-Human HRP Detection System	Manual IHC (Biocare Hs-on-Hs-A01)

**See next page for protocol details*

Protocol Details

Protocol ID: Manual IHC (Biocare Hs-on-Hs-A01)

Pre-processing (offline)

Step	Temperature	Time
Baking	60ºC	30 min
Xylene	Ambient	5 min
Xylene	Ambient	5 min
Xylene	Ambient	5 min
Xylene	Ambient	5 min
100% Ethanol	Ambient	30 sec
100% Ethanol	Ambient	30 sec
Air dry	Ambient	Until dry

Epitope Retrieval (offline)

Step	Temperature	Time
**DIVA Decloaker Solution (1X, RTU)**	Ambient	0
**DIVA Decloaker Solution (1X, RTU)**	110ºC	Varies
dH2O	Ambient	0
dH2O	0ºC	20 min
TBS/T	Ambient	0
TBS/T	Ambient	0
**Peroxide Block 3%**	Ambient	10 min
TBS/T	Ambient	0
TBS/T	Ambient	0
TBS/T	Ambient	7 min
TBS/T	Ambient	7 min

Staining (offline)

Step	Temperature	Time
**Serum Block, RTU (Invitrogen cat# 37527)**	Ambient	30 min
TBS/T	Ambient	0
TBS/T	Ambient	0
TBS/T	Ambient	7 min
TBS/T	Ambient	7 min
**Primary Antibody (DIG Conj. + Hs Abs)**	Ambient	60 min
TBS/T	Ambient	0
TBS/T	Ambient	0
TBS/T	Ambient	7 min
TBS/T	Ambient	7 min
**Mouse anti-Digoxigenin Secondary (BRR4055G)**	Ambient	30 min
TBS/T	Ambient	0
TBS/T	Ambient	0
TBS/T	Ambient	7 min
TBS/T	Ambient	7 min
**MACH 2 Mouse HRP-Polymer (MHRP520G)**	Ambient	30 min
TBS/T	Ambient	0
TBS/T	Ambient	0
TBS/T	Ambient	7 min
TBS/T	Ambient	7 min
dIH2O	Ambient	0
**DAB (CST 1:50)**	Ambient	0
**DAB (CST 1:50)**	Ambient	5 min
dIH_2O	Ambient	0
dIH_2O	Ambient	0
dIH_2O	Ambient	0
dIH_2O	Ambient	0
dIH_2O	Ambient	0

Post-processing (offline)

Step	Temperature	Time
Hematoxylin (Gill 1)	Ambient	15 sec
Tap H2O rinse	Ambient	0
Bluing (0.5% NaHCO3)	Ambient	30 sec
Tap H2O rinse	Ambient	0
dH2O rinse	Ambient	0
dH2O rinse	Ambient	0
70% Ethanol	Ambient	30 sec
100% Ethanol	Ambient	30 sec
100% Ethanol	Ambient	30 sec
100% Ethanol	Ambient	30 sec
Xylene	Ambient	30 sec
Xylene	Ambient	30 sec
Xylene	Ambient	30 sec
Cytoseal XYL mount		
