# Supplementary material for: Affinity Maturation and Characterization of the Novel Monoclonal Antibody (mAb) PB-223 Targeting Cancer-Specific O-Glycans Terminating with α(2,6) Sialic Acids
Source: Cancers (Basel). 2026 Jul 20;18(14):2336. doi: 10.3390/cancers18142336 (PMC13406998; doi:10.3390/cancers18142336)
Supplement: Supplementary file 1 [file cancers-18-02336-s001.zip › Supplemental Figure S2.pdf]

Supplemental Figure S2. Monoisotopic mass, proposed compositions, proposed structures, and relative abundance of the O-glycan expressed by each cell line tested.

m/z	Proposed Composition	Proposed Structure	Relative Intensity HCC-1937	Relative Intensity LOVO	Relative Intensity OV-90
749.3	(Hex)2(HexNAc)2	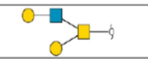	0.01%	0.02%	0.14%
837.3	(Hex)2(HexNAc)1(NeuAc)1 A	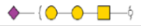	2.28%	0.22%	0.38%
837.3	(Hex)2(HexNAc)1(NeuAc)1 B	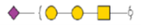	3.45%	0.03%	0.05%
837.3	(Hex)2(HexNAc)1(NeuAc)1 C	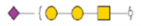	0.02%	0.34%	0.14%
675.2	(Hex)1(HexNAc)1(NeuAc)1	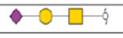	14.47%	28.02%	21.01%
546.2	(Hex)2(HexNAc)1 A	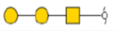	0.13%	0.08%	2.34%
546.2	(Hex)2(HexNAc)1 B	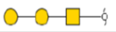	0.13%	0.06%	0.78%
790.3	(Hex)1(HexNAc)3	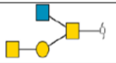	0.10%	0.06%	0.12%
530.2	(Hex)1(HexNAc)1(Deoxyhexose)1	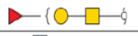	0.11%	0.11%	0.19%
587.2	(Hex)1(HexNAc)2	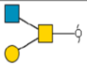	0.01%	0.07%	0.15%
895.3	(Hex)2(HexNAc)2(Deoxyhexose)1 A	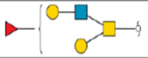	0.31%	0.21%	0.65%
895.3	(Hex)2(HexNAc)2(Deoxyhexose)1 B	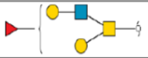	0.04%	0.01%	0.97%
966.3	(Hex)1(HexNAc)1(NeuAc)2	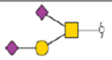	54.88%	62.15%	49.43%
1260.5	(Hex)3(HexNAc)3(Deoxyhexose)1	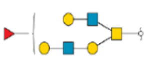	2.72%	0.24%	2.21%
1098.4	(Hex)2(HexNAc)3(Deoxyhexose)1	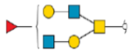	0.00%	0.00%	0.22%
1057.4	(Hex)3(HexNAc)2(Deoxyhexose)1	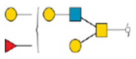	0.21%	0.16%	0.44%
1331.5	(Hex)2(HexNAc)2(NeuAc)2	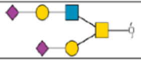	7.42%	3.09%	4.36%
1040.4	(Hex)2(HexNAc)2(NeuAc)1	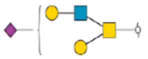	6.32%	0.29%	6.76%
691.2	(Hex)1(HexNAc)1(NeuGc)1	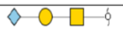	0.04%	0.28%	0.24%
821.3	(Hex)1(HexNAc)1(Deoxyhexose)1(NeuAc)1	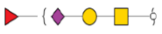	0.76%	0.24%	0.94%
878.3	(Hex)1(HexNAc)2(NeuAc)1 A	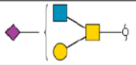	4.26%	3.01%	5.31%
878.3	(Hex)1(HexNAc)2(NeuAc)1 B	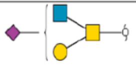	2.34%	1.32%	3.18%

Monosaccharide Key:
: Galactose
: Fucose
: *N*-acetyl glucosamine (GlcNAc)
: Mannose
: Pentose
: *N*-glycolyl neuraminic acid (NeuGc)
: *N*-acetyl neuraminic acid (NeuAc)
: *N*-acetyl hexosamine (HexNAc, undefined stereochemistry)
: Hexose (Hex, undefined stereochemistry)
P : Phosphate (Phos)
S : Sulfated (Sulph)
