# Supplementary figures and images for: Affinity Maturation and Characterization of the Novel Monoclonal Antibody (mAb) PB-223 Targeting Cancer-Specific O-Glycans Terminating with α(2,6) Sialic Acids
Source: Cancers (Basel). 2026 Jul 20;18(14):2336. doi: 10.3390/cancers18142336 (PMC13406998; doi:10.3390/cancers18142336)

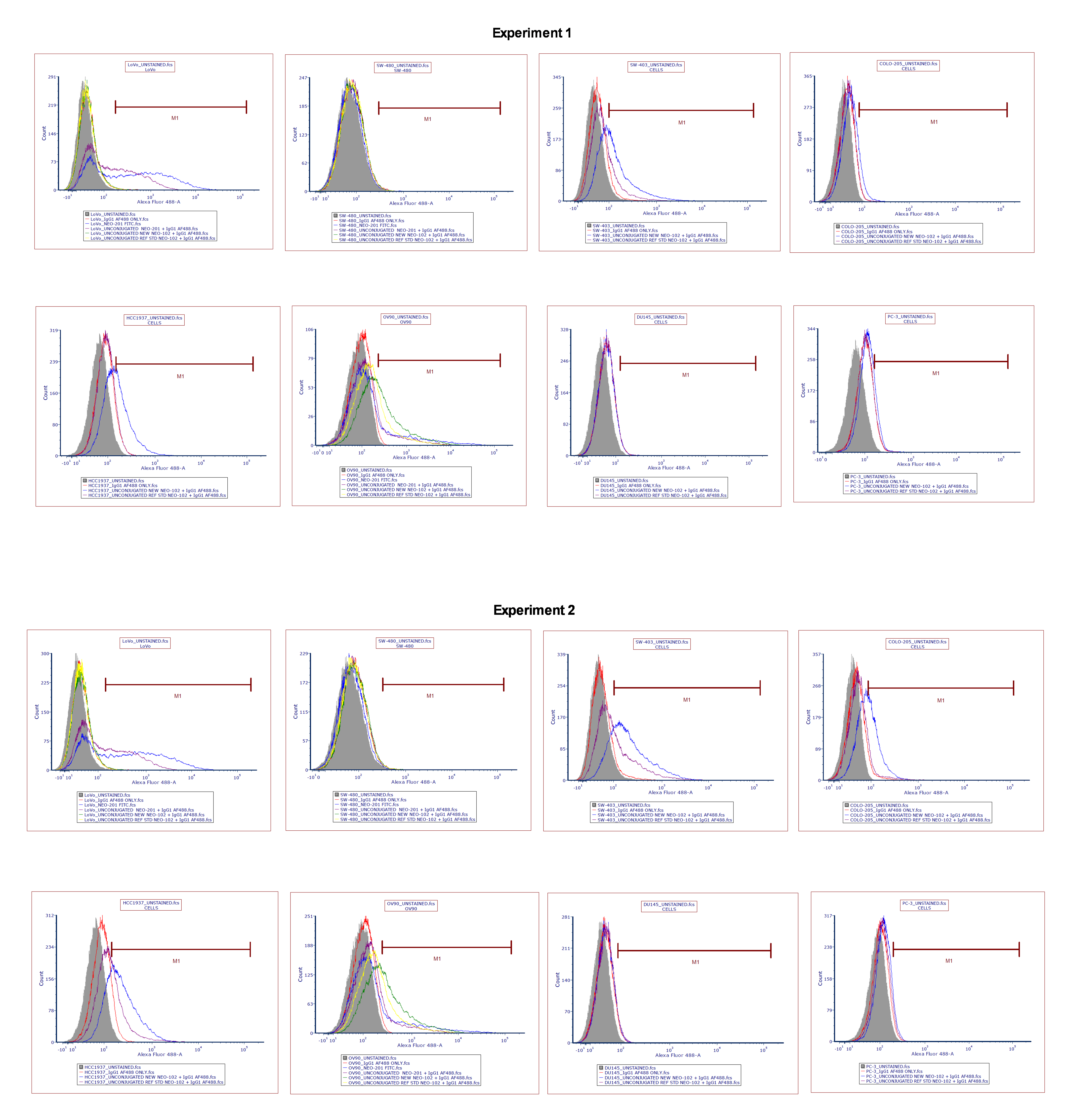

Supplement: Supplementary file 1 [file cancers-18-02336-s001.zip › Supplemental Figure S1.tif]
